# A Nitrocellulose Paper-Based Multi-Well Plate for Point-of-Care ELISA

**DOI:** 10.3390/mi13122232

**Published:** 2022-12-16

**Authors:** Zhen Qin, Zongjie Huang, Peng Pan, Yueyue Pan, Runze Zuo, Yu Sun, Xinyu Liu

**Affiliations:** 1 Department of Mechanical and Industrial Engineering, University of Toronto, Toronto, ON M5S 3G8, Canada; 2 Institute of Biomedical Engineering, University of Toronto, Toronto, ON M5S 3G8, Canada

**Keywords:** paper-based ELISA, nitrocellulose, multi-well paper plate, point-of-care diagnostics

## Abstract

Low-cost diagnostic tools for point-of-care immunoassays, such as the paper-based enzyme-linked immunoassay (ELISA), have become increasingly important, especially so in the recent COVID-19 pandemic. ELISA is the gold-standard antibody/antigen sensing method. This paper reports an easy-to-fabricate nitrocellulose (NC) paper plate, coupled with a desktop scanner for ELISA, which provides a higher protein immobilization efficiency than the conventional cellulose paper-based ELISA platforms. The experiments were performed using spiked samples for the direct ELISA of rabbit IgG with a limit of detection (LOD) of 1.016 μg/mL, in a measurement range of 10 ng/mL to 1 mg/mL, and for the sandwich ELISA of sperm protein (SP-10) with an LOD of 88.8 ng/mL, in a measurement range of 1 ng/mL to 100 μg/mL. The described fabrication method, based on laser-cutting, is a highly flexible one-step laser micromachining process, which enables the rapid production of low-cost NC paper-based multi-well plates with different sizes for the ELISA measurements.

## 1. Introduction

**Table of Contents:** A nitrocellulose paper-based multi-well plate provides high protein immobilization efficiency and is used for rapid enzyme-linked immunosorbent assays (ELISAs).

Paper-based microfluidic devices have attracted increasing attention and are widely applied to analytical applications for point-of-care (POC) diagnostics [1], including the ones for COVID-19 testing [2,3,4,5]. Paper substrates share common advantages, such as a good biocompatibility with biomolecules (e.g., proteins and nucleic acids) and pump-free fluid transport through the capillarity [6,7]. The paper-based devices are compatible with various quantitative detection mechanisms, such as colorimetry [8,9], electrochemistry [10,11,12], chemiluminescence [13], and electrical conductivity [14]. Among them, the colorimetric enzyme-linked immunosorbent assay (ELISA) on a paper-based device, is of great potential. The colorimetric output signal of a paper-based ELISA can be readily quantified through low-cost and simple instruments, such as a digital camera, a smartphone, or a portable scanner [15].

The paper-based colorimetric ELISA was first demonstrated by Cheng et al., through a wax-printed paper-based multi-well plate [16]. It has been extended to the detection of human performance biomarkers [17], bullous pemphigoids [18], tumor markers [19], bacteria [20], T7 bacteriophages [21], cancer biomarkers [22] and recently, the SARS-CoV-2 antibody and antigen [23,24]. The paper substrate adopted in these studies was mainly pure cellulose filter/chromatography paper. However, a distinct drawback of the pristine cellulose paper is its low protein immobilization efficiency [25]. The cellulose paper substrate usually needs to be pre-treated with organic linkers, to enhance the paper’s protein binding affinity (through covalent protein binding), and thus improves the analytical performance of the paper-based ELISA [26,27,28]. 

Compared to the widely investigated cellulose paper, the paper-like nitrocellulose (NC) membrane (or simply “NC paper”), made from pure cellulose nitrate, possesses more uniform porous structures and a larger surface-area-to-volume ratio [29]. The hydrophobic interaction between the carbon-containing NC and the hydrophobic portion of the protein empowers the NC paper with a better protein-binding capacity [30]. The NC paper has been used as the substrate material in the lateral flow immunochromatographic assays (LFIAs), and the capture proteins for the test and control lines can be directly plotted on the NC paper, without any chemical treatment of the NC surface [31,32]. Although featuring a high protein-binding capability, the NC paper is less applied as a microfluidic paper-based device, apart from lateral flow strips, due to the challenge of generating robust hydrophobic walls on the NC membranes. It is also difficult, on NC paper, to realize the origami type of the structure, such as the origami cellulose paper device, due to its fragile nature [33]. The NC paper-based microfluidic devices have been mainly developed for immunoassays in a two-dimensional chip format [34,35]. 

Multiple techniques have been reported for the fabrication of paper-based microfluidic devices [36], including photolithography [37,38], inkjet printing [39], wax printing [40,41], and also laser-cutting [42,43]. However, not all of these methods are suitable for fabricating NC paper-based devices. Creating hydrophobic walls through wax printing on NC paper, is challenging, because NC paper is highly flammable and brittle. Although wax-printing on NC paper has been proven to be feasible [44], the step of high-temperature baking involved can damage the pore structure, and the wax barrier can be breached by the bioanalysis solutions. As an alternative to wax printing, a recent study applies screen-printed polyurethane acrylate (PUA) as the barrier material and UV-curing to define the flow channel and reaction zone on NC paper [45]. The photolithography method requires sophisticated equipment and expensive photoresists, and involves multi-steps for the device fabrication. A NC paper device can also be fabricated through manual punch, but is limited in puncher designs [46]. In contrast, laser cutting is a simple way to pattern a NC membrane into designed shapes and is a promising candidate for a NC paper-based device fabrication [47]. 

Although NC paper has been widely used for LFIAs, no attempt has been made to fabricate NC paper-based multi-well plates, for a colorimetric ELISA. We hypothesize that the excellent protein immobilization capability of the NC paper could improve the sensitivity of the colorimetric ELISA. In this paper, we fabricated a NC paper-based multi-well plate through a simple laser-cutting method, and applied the multi-well plate to conduct the colorimetric ELISA of the human sperm protein 10 (SP-10, a protein marker to quantify the sperm numbers in human semen) [48]. To characterize the analytical performance of the NC paper-based multi-well plate, we first conducted a direct ELISA of rabbit IgG, and experimentally optimized the assay parameters. The colorimetric signal was quantified with a desktop scanner. The results show that the direct ELISA of rabbit IgG on NC paper provides a higher analytical sensitivity than that of the same assay on cellulose paper. Then, we carried out a sandwich ELISA of SP-10 and achieved a LOD of 88.8 ng/mL. This paper demonstrates the improved analytical performance of a NC paper-based ELISA, due to the superior protein immobilization capability of the NC substrate. The developed NC paper-based multi-well plate can serve as a low-cost platform for ELISA-based diagnostics.

## 2. Materials and Methods

### 2.1. Design and Fabrication of the NC Paper-Based Multi-Well Plate 

**Figure 1:**
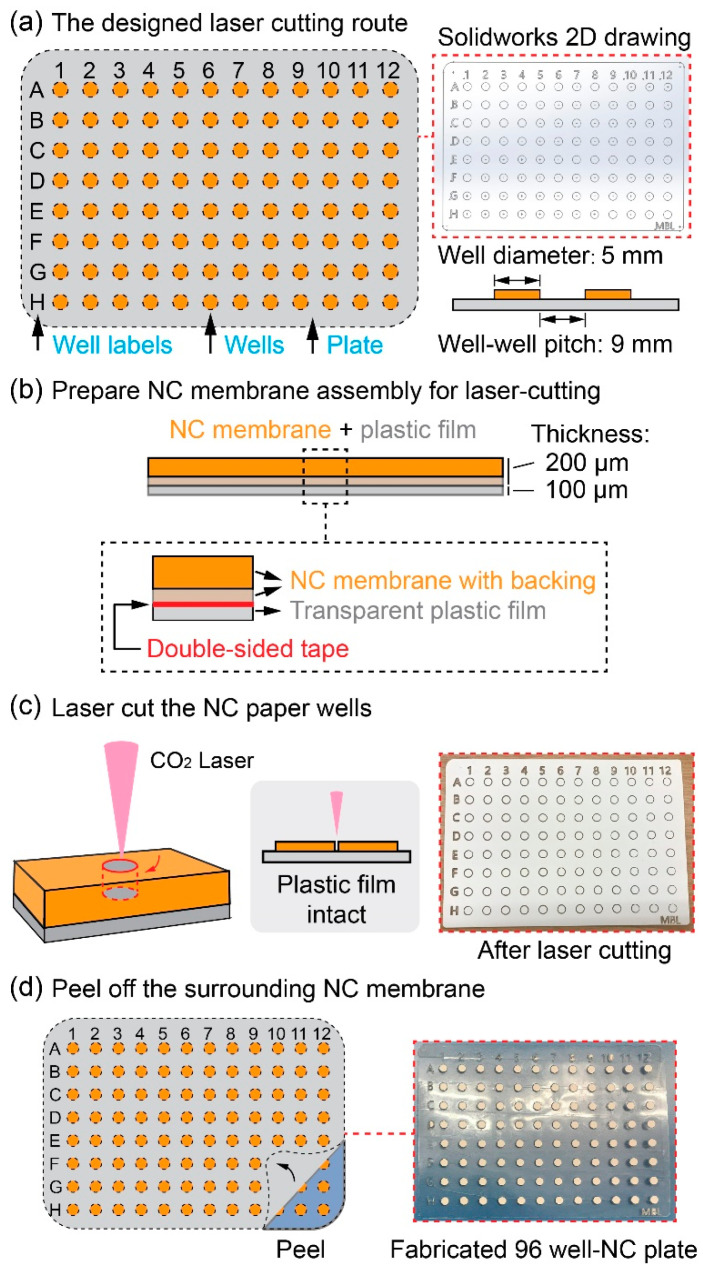
Fabrication of the NC paper-based 96-well plate through a simple laser-cutting method. (**a**) Design of the laser-cutting patterns of the 96-well plate. (**b**) The assembly of the NC paper (including its plastic backing) with the plastic film. (**c**) The laser-cutting and (**d**) peel-off processes of the NC paper surrounding the NC wells.

To fabricate the NC paper-based multi-well plate, a 96-well plate pattern (well diameter: 5 mm, and well-center pitch: 9 mm) was first generated in Solidworks 2017, as shown in Figure 1a. Prior to the laser cutting process, a piece of NC paper (GE Health #FF80HP, Cytiva), composed of a 200 µm NC membrane and a 100 µm polyester backing, was attached to a copier transparency film (Staples), with the assistance of a double-sided adhesive tape (Figure 1b). The assembly of the NC paper and the plastic film was then placed in a CO_2_ laser cutter (Universal Laser System: VLS-desktop-model-350), and the assembly was laser-cut following the pre-defined route (Figure 1c). The laser-cutting parameters, including the laser power intensity and the cutting speed, were optimized to make the top-layer NC membrane of the assembly be cut through, while the bottom-layer plastic film remained intact, as illustrated in the inset figure in Figure 1c. The optimized operation settings of the laser cutter were 10% power, 100% speed, and 1000 PPI (PPI: laser pulse per inch). These laser cutting parameters ensured a fast and precise cutting of the NC membrane, without obvious material burning (due to overheating). In the laser cutting experiments, it was observed that the laser cutting fused the edges of the NC-paper-disc plastic backing with the transparent plastic film at the bottom of the assembly. Thus, the leftover NC paper discs were able to strongly adhere to the plastic film because of both the double-sided tape and the fusion effect induced by the laser cutting. The NC membrane piece surrounding the NC wells was lastly peeled off and the final 96-well NC paper plate was formed (Figure 1d).

### 2.2. Direct ELISA Protocol for the Rabbit IgG Detection

**Figure 2:**
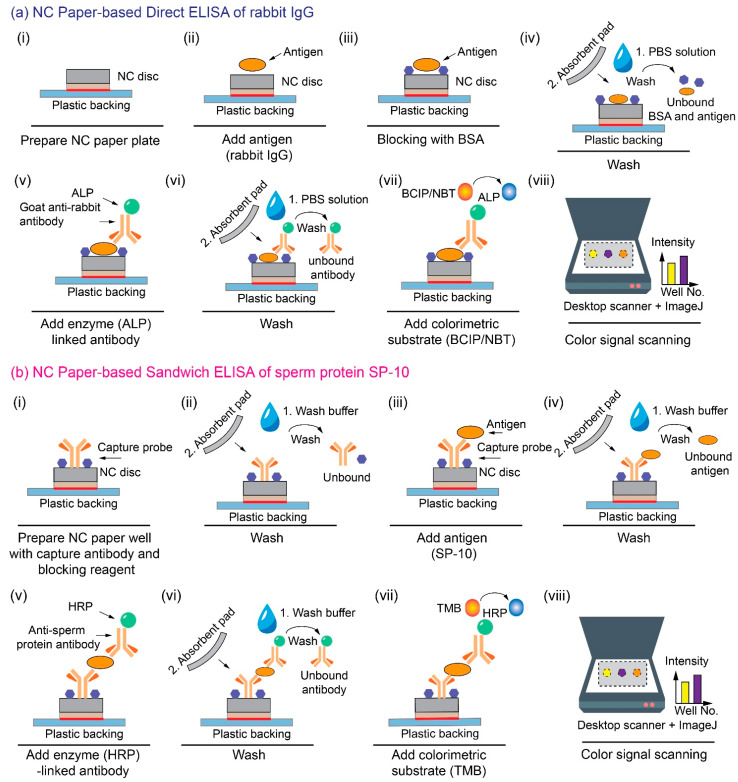
The schematic representation of the (**a**) direct ELISA of rabbit IgG and (**b**) the sandwich ELISA of sperm protein SP-10 on the NC paper multi-well plate.

To characterize the analytical performance of the NC paper-based 96-well plate for the ELISA, we first performed a direct ELISA of rabbit IgG antigen in a phosphate-buffered saline (PBS) solution (cat #10010023, Gibco). The purity and concentrations of the rabbit IgG reagents were quantified by the supplier and thus we assumed the spiked samples’ concentrations were as we expected. The detailed direct ELISA protocol on the NC paper multi-well plate is described below:

- As shown in Figure 2(a-i,a-ii), the direct ELISA of rabbit IgG starts with the addition of 3 μL of rabbit IgG (cat #I5006, Sigma-Aldrich) in a PBS solution onto each NC well, followed by a 10-min incubation at room temperature,
- Then, 3 μL of 4% bovine serum albumin (BSA; cat #05482, Sigma-Aldrich) solution was added to block the vacant binding sites on the NC well, to minimize the non-specific binding (Figure 2(a-iii)), and the blocked NC well was incubated at room temperature for 10 min,
- Next, the NC well was washed with 5 μL of 1× PBS buffer three times. The wash buffer was absorbed each time with an absorbent paper (cat #WHA10427806, Cytiva), and the washed NC well was finally incubated for 10 min at room temperature (Figure 2(a-iv)),
- Three μL of the ALP-conjugated rabbit IgG antibody (cat #A3687, Sigma-Aldrich) solution, diluted from the stock solution at the ratio of 1:1000, was added onto each NC well for 10-min incubation (Figure 2(a-v)),
- Finally, each NC well was washed with 5 µL of 1× PBS solution three times (Figure 2(a-vi)), and 4 μL of BCIP/NBT (5-bromo-4-chloro-3′-indolyphosphate p-toluidine salt/nitro-blue tetrazolium, cat #B5655, Sigma-Aldrich) substrate was deposited onto each NC well (Figure 2a-vii). The colorimetric signal was quantified after a 10-min incubation (Figure 2(a-viii)). The whole direct ELISA of rabbit IgG takes around 1 h to complete.

In comparison, the cellulose paper-based ELISA applied the following protocol:

- First, the wax-printed cellulose paper well was washed with 30 μL PBS and dried in the air for 15 min,
- Second, 5 μL of the rabbit IgG (cat #I5006, Sigma-Aldrich) in the PBS solution was added to a cellulose paper well as an antigen. Five μL of the blank PBS solution was added to a separate well as the control. A 20-min incubation was followed to allow the wells to dry in the air,
- Third, 5 μL of the blocking buffer composed of 2% w/v BSA (cat #05482, Sigma Aldrich) and 0.05% w/v Tween 20 (cat #P1379, Sigma Aldrich) in PBS, was added to the test wells, followed by a 30-min incubation,
- Fourth, 5 μL of the ALP-conjugated rabbit IgG antibody solution (cat #A3687, Sigma Aldrich, 1: 500 diluted in PBS) was added to the well,
- Fifth, 10 μL of PBS was added to the wells and afterwards absorbed with a piece of the adsorbent pad (cat #28297-998, VWR) three times,
- Sixth, 8 μL of BCIP/NBT substrate was added to each well for the color change with an incubation time of 30 min,
- Lastly, the dried cellulose paper plate was placed in the scanner for the color intensity analysis.

### 2.3. Sandwich ELISA Protocol for the SP-10 Detection

We also performed the sandwich ELISA of SP-10 on the NC paper multi-well plate. The reagents are from the human ACRV1 ELISA pair set (cat #SEK11789, Sino Biological). The sandwich ELISA protocol is described as follows:

- The sandwich ELISA of SP-10 starts with the addition of 3 μL of the capture antibody solution (mouse anti-ACRV1 monoclonal antibody in CBS solution, cat #11789-MM01, Sino Biological) with a concentration of 100 μg/mL, onto the NC paper well, followed by the overnight incubation in a 4 °C fridge for the sufficient antibody immobilization,
- Then, after taking the blocked plate out of the fridge, each NC paper well was pipetted with 10 μL of the wash buffer (0.05% Tween20 in TBS, pH 7.2–7.4) and blotted with an absorbent pad, three times, to remove the unbound antibodies,
- Next, 3 μL of the blocking buffer (2% BSA in wash buffer) was added to each well (Figure 2(b-i)), followed by a 10-min incubation and washing step with 10 μL wash buffer, three times, again to remove the unbound blocking reagents (Figure 2(b-ii)),
- Next, 3 μL of the SP-10 protein solution (cat #10227-H08B, Sino Biological) was added to the blocked NC paper well and incubated for 10 min, for the SP-10 protein to be captured by the immobilized capture antibody probe (Figure 2(b-iii)). The well was also washed with 10 μL of wash buffer, three times, to prevent the non-specific binding of the antigen (Figure 2(b-iv)),
- Next, 3 μL of the detection horseradish peroxidase (HRP) conjugated SP-10 antibody (0.1 μg/mL, cat #11789-MM06, Sino Biological) was added to each well and incubated for 10 min (Figure 2(b-v)). The well was sequentially washed three times with 10 μL of wash buffer to remove the non-specific binding of the signal antibodies (Figure 2(b-vi)),
- Lastly, 5 μL of the TMB (3,3’,5,5’-tetramethylbenzidine) substrate (10 mg/mL in DMSO) was added to each well and incubated for 5 min for the color generation (Figure 2(b-vii)). The dried NC paper plate was placed in the scanner for analysis (Figure 2(b-viii)). The whole sandwich ELISA of sperm protein on the NC paper takes around 50 min to complete.

### 2.4. Colorimetric Signal Interpretation with a Desktop Scanner 

For the colorimetric signal readout, a desktop scanner (Canon LiDE 210) was used to scan the 96-well plate into a digital RGB image, and the software ImageJ (NIH) was used to quantify the grayscale intensity of the colorimetric signal in each well post ELISA reactions. During the image analysis in ImageJ, a circle was drawn along the NC paper well boundary in the scanned plate image, as the analysis area. In this study, the direct ELISA of rabbit IgG generates a purple color signal through the reaction of the alkaline phosphate (ALP) enzyme label and its BCIP/NBT substrate, while the sandwiched ELISA of SP-10 produces a blue color signal through the reaction of the HRP enzyme label and its TMB substrate. Each well’s mean greyscale intensity (the average of the red, green, and blue channel intensities) is quantified through ImageJ. All of the reported results were carried out in replicates, to ensure the result’s accuracy and repeatability. The number of replicates is indicated by the variable N. 

## 3. Results and Discussion

### 3.1. Direct ELISA Protocol Optimization

**Figure 3:**
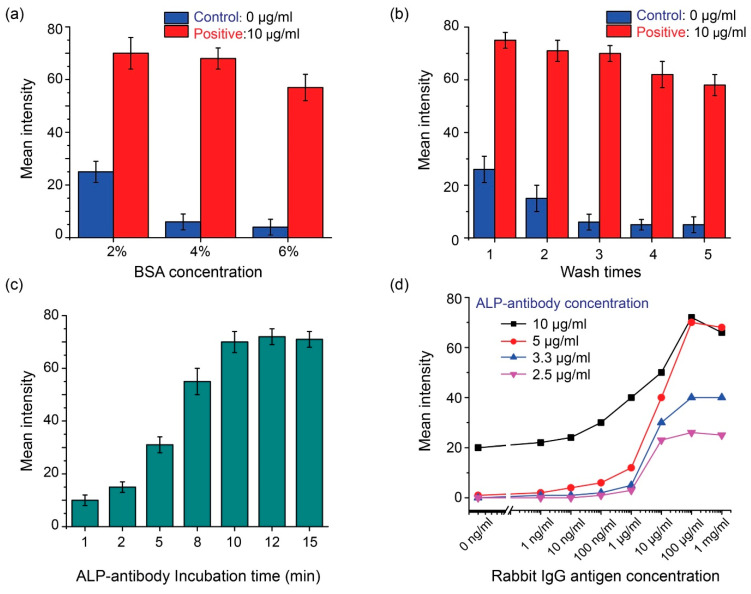
The direct ELISA optimization of the assay protocol parameters, including (**a**) the BSA concentration, (**b**) number of washes with the PBS solution, (**c**) incubation time after adding the ALP-conjugated antibody, and (**d**) the applied ALP-antibody concentration (through changing the dilution factor of the stock solution).

The direct ELISA protocol, described in Section 2.2, is with the optimized parameters. The optimization of the protocol parameters is described below. Using 10 μg/mL of rabbit IgG as the positive sample, the effect of the BSA concentration for blocking was first experimentally examined. As shown in Figure 3a, the results suggest that 4% BSA is effective for minimizing the non-specific binding of the control group samples and in the meanwhile maintaining a high signal output for the positive group samples. Further increasing of the BSA concentration to 6%, reduced the signal output, due to the over-blocking of the target rabbit IgG antigen. Thus, the BSA concentration was selected to be 4%. 

Efficient washing after the addition of the ALP-conjugated goat rabbit IgG antibody is necessary, to remove the non-specific binding and thus reduce the background noise; thus, the effect of the PBS washing times (with a fixed washing volume of 5 µL of 1× PBS) was tested and the results were shown in Figure 3b. One or two washes caused a high background noise in the control group, representing inadequate washing. Three washes were found to be sufficient, and the signal intensity of the control group was maintained at a low level. Washing more than three times did not further reduce the background noise level of the control group, but significantly reduced the signal output level of the positive group samples. Thus, three washes were selected as the final washing parameter. 

The ALP-conjugated antibody was added to the NC well for binding with the IgG antigen immobilized, and eventually produced a colorimetric signal indicating the amount of the IgG antigen. The incubation time of the NC well, after the addition of the ALP-conjugated antibody, was investigated. All the other protocol parameters remained the same as the optimized protocol described in Section 2.2. As shown in Figure 3c, the output signal intensity increased with the incubation time and remained stable when the incubation time was 10 min and above. Thus, a 10-min incubation was adopted in the final protocol for the colorimetric signal production. 

A proper concentration of the ALP-conjugated antibody is also important, as it directly affects the signal production. A too-low antibody concentration could induce an insufficient number of antibodies for labelling the target antigen, and a very high antibody concentration could yield an excessive number of antibodies that may boost non-specific antibody binding, and thus the background noise level. The experimental results in Figure 3a–c confirmed the stability of the developed NC-paper-based ELISA performance. Thus in Figure 3d, we applied the one-time screening to find the optimal ALP-antibody concentration that gives the best sensitivity. Here we applied different concentrations of the ALP-antibody for the direct ELISA, by changing the stock solution (5 mg/mL) dilution to 10 μg/mL (dilution ratio of 1:500) to 2.5 μg/mL (dilution ratio of 1:2000), to find the optimal one for the rabbit IgG detection protocol. Based on the result in Figure 3d, the background noise, due to the non-specific binding, would be higher than in the other groups, when the antibody concentration applied is 10 μg/mL. A higher LOD was achieved for the device with an excessively high antibody dilution ratio. When the too-low antibody concentrations of 3.3 μg/mL (dilution ratio of 1:1500) and 2.5 μg/mL were used, the signal level or the sensitivity was lower than the 5 μg/mL (dilution ratio of 1:1000), thus limiting the linear detection range of the device. Following the screening, we determined that the optimal concentration of the ALP-antibody solution to be applied in the protocol was 5 μg/mL.

### 3.2. Calibration Results of the Direct ELISA of Rabbit IgG

**Figure 4:**
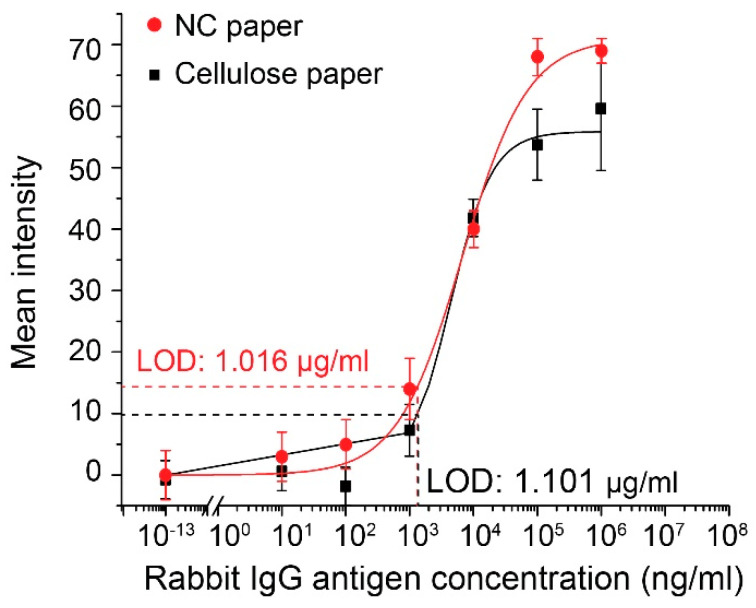
Calibration plot for the mean grayscale intensity of the color produced by the enzymatic reaction in the direct ELISA versus the amount of rabbit IgG absorbed into each well of the cellulose and NC paper-based multi-well plates. Each data point is the mean of eight replicates (N = 8) and the error bars represent the standard deviations of the measurements.

The direct ELISA was conducted for the detection of the antigen of rabbit IgG in 10-fold dilutions, from the concentration of 10 ng/mL to 1 mg/mL. The colorimetric signal intensity of each well measured by the scanner, was darker as the antigen amount increased. The average pixel intensity value for each well was analyzed through the ImageJ software. A sigmoidal curve (Figure 4) was generated by the non-linear regression using the Hill equation, which shows a high correlation between the signal intensity and the sample concentration (R^2^ = 0.992). The LOD achieved with the optimized protocol was 1.016 μg/mL, as determined following the three-sigma limit to be three standard deviations from the mean intensity of the negative control. The observed linear range was from 1 μg/mL to 100 μg/mL. The calibration curve for the direct ELISA on a cellulose paper plate was also shown in Figure 4 (the black curve). One can see that at low concentrations (10 ng/mL and 100 ng/mL), the NC paper plate provides a greater signal intensity than the cellulose paper plate. This is due to the higher protein capture efficiency of the NC paper than the cellulose paper. The assay on the cellulose paper plate yields an LOD of 1.101 μg/mL, which is also slightly higher than that (1.016 μg/mL) of the NC paper plate. The direct ELISA on cellulose paper was also optimized similarly, and showed a similar performance, compared to the previous literature [16]. We believe that there is still room for us to further improve the LOD of the direct ELISA on the NC paper plate, which can be realized through the optimization of the washing buffer composition for the enhanced washing efficiency.

### 3.3. Calibration Results of the Sandwich ELISA of SP-10

**Figure 5:**
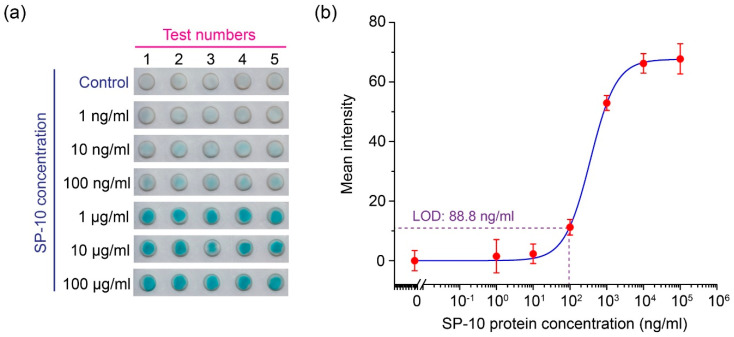
The sperm protein sandwich ELISA detection on the NC paper plate. (**a**) The experimental photo of the sperm protein sandwich ELISA on the NC paper plate (N = 5), and (**b**) the grayscale intensity scanned by the desktop scanner and the generated calibration curve, based on the Hill equation.

The sandwich ELISA, for detecting the SP-10, was conducted and the calibration results are shown in Figure 5. Figure 5a presents the NC paper-based ELISA colorimetric outputs at different concentrations of SP-10, as inputs ranging from 1 ng/mL to 100 μg/mL, plus a control group. Figure 5b shows the signal intensity calibration curve achieved from scanning the NC paper well color intensities with the desktop scanner. The LOD of the sandwich ELISA assay, following the protocol described in Section 2.3, was determined to be 88.8 ng/mL. 

The proposed laser-cutting method for the NC paper plate fabrication has a high degree of flexibility for creating different sizes of NC paper plates. The 96-well plate format is demonstrated in this study to be feasible, since the 96-well plate is the most used plate type for the bioassays. Other different sizes of NC paper wells can be prepared by using the proposed laser-cutting method. The fluid operations on the NC paper plate, such as the incubation of antibodies or washing the wells, can be easily realized through a single-channel pipette or a multi-channel pipetting/automated robotic handling, which is more efficient. The NC-paper-plate-based ELISA is also compatible with a plate reader for the accurate absorbance-based colorimetric reading. The current assay applied a desktop scanner and the computer software ImageJ for the signal quantification. For the POC applications, the future development can incorporate a smartphone camera to read the signal, to improve the portability. The developed NC paper-based plate provided LODs of 1.016 μg/mL for the direct ELISA and 88.8 ng/mL for the sandwich ELISA. Compared to the standard ELISA on a plastic multi-well plate for SP-10, the NC paper-based ELISA uses much lower volumes of samples and reagents (Table 1). Moreover, the proposed NC paper-based ELISA also has other advantages, including a shorter assay time (<1 h for the NC paper-based ELISA, compared to several hours for a standard 96-well plate-based ELISA), a lower cost for the plate fabrication, and an easier signal quantification method.

## 4. Conclusions

This work developed a NC paper-based 96-well plate that can be produced with a simple and rapid one-step laser-cutting method. The surface property of the NC paper renders it more efficient for the protein immobilization, than cellulose paper, thus enhancing the assay sensitivity on the NC paper-based plate. The developed NC 96-well plate is compatible with standard laboratory instruments for liquid handling and signal readout. For proof of demonstration, the direct ELISA of rabbit IgG and the sandwich ELISA of SP-10 were successfully performed, and the LODs of 1.016 μg/mL and 88.8 ng/mL were achieved, respectively. 

## Figures and Tables

**Table 1:** The comparison between the SP-10 NC-paper-based ELISA and the standard plate-based ELISA for the SP-10 detection.

Compared Properties	NC Paper-Based Sandwich ELISA	Standard Plate-Based ELISA
Sample size	3 μL	100 μL
2. Total reagent consumed	~137 μL	450 μL
3. Total assay time	~ 50 min	>3.5 h
4. Detection limit	88.8 ng/ml	46.875 pg/ml
5. SP-10 protein biomarker detectable weight	266.4 pg	4.6875 pg
6. Signal detection	Low-cost desktop scanner (~70 CAD)	Expensive microplate reader (~100k CAD)

## Data Availability

The datasets generated during and/or analysed during the current study are available from the corresponding author on reasonable request.
